# Comparison of the Short- and Long-Term Effects of Radiofrequency Thermocoagulation and Intralesional Cryoablation Treatments on Pain Management and Functional Recovery in Patients Diagnosed With Calcaneal Spur: Retrospective Study

**DOI:** 10.1155/prm/4521963

**Published:** 2025-10-15

**Authors:** Ahmet Yilmaz

**Affiliations:** Department of Anesthesiology & Reanimation, Discipline of Pain Medicine, Ministry of Health Adana City Training & Research Hospital, Adana, Turkey

**Keywords:** AOFAS, cryoablation, functional improvement, pain management, radiofrequency

## Abstract

**Objectives:**

Calcaneal spur is an important cause of chronic pain that is resistant to conservative treatments and reduces quality of life. Nerve ablation techniques are widely used in chronic pain. This study aimed to investigate the short- and long-term effects of radiofrequency thermocoagulation (RFT) and intralesional cryoablation (CA) on pain management, functional limitation, and ankle function in patients with calcaneal spurs.

**Materials and Methods:**

This study included 71 patients aged between 28 and 52 years (body mass index, 22.3–31.1) who were receiving chronic pain treatment. The patients were evaluated pre- and postoperatively using the visual analog scale, functional limitation, activity limitation, and American Orthopedic Foot and Ankle Society score.

**Results:**

VAS was measured as 1.2 ± 0.1 in the RFT group and 1.3 ± 0.2 in the CA group at 6 months. RFT yielded faster and more effective results in pain management and short-term functional improvement (*p*=0.00002), whereas CA was superior to the former in terms of ankle function in the long term (*p*=0.0001).

**Conclusion:**

RFT and CA have different advantages. Thus, personalized treatment should be provided to each patient. Although our study was conducted retrospectively, prospective studies are needed to support our findings.

## 1. Introduction

Heel pain is a frequent complaint in the algology clinic and is caused by different etiological factors [1]. It may occur due to nerve damage, trauma, degenerative diseases, etc., and limits the patient's daily activities, resulting in physical and psychological difficulties [2]. One of the most common causes of heel pain is a calcaneal spur [3]. The calcaneal spur originates from the calcaneal tubercle and is usually located in the medial part. It is generally triangular in shape and has a pointed tip. The variable regions of the spur are enveloped by a fibrous connective tissue layer that is highly vascularized and densely innervated [4].

Conservative approaches constitute the initial stage of treatment. These include rest, the use of soft-soled footwear, orthotic insoles, therapeutic exercises, and nonsteroidal anti-inflammatory medications. For patients who fail to respond adequately to these measures, alternative interventions such as corticosteroid or platelet-rich plasma injections, various physical therapy techniques, and extracorporeal shock wave therapy are frequently applied [5]. However, patients whose pain persists despite all these treatments do not always accept surgery as a treatment alternative and continue to seek treatment.

Among the methods employed in the treatment of chronic pain, minimally invasive techniques, such as radiofrequency (RF) and cryoablation (CA), offer an important solution for patients. RFT and CA have low complication rates and are effective in pain palliation [5]. These treatment modalities are easily applied and allow patients to return to their daily activities the same day. For these reasons, we preferred these methods for patients with calcaneal spurs resistant to conservative treatments.

RFT can be used to neutralize the transmission of pain signals by heating the lesion in the tissues. Herein, RFT was used owing to its easy and short application technique.

CA involves in situ destruction of the lesion in painful tissues via freezing. Ultrastructural studies reported that ice crystals are formed inside the cell and that the resulting cell damage is osmotic and secondary in terms of the chronological development of tissue necrosis [2].

RFT provides rapid pain relief by targeting pain transmission [6]. Meanwhile, CA is aimed at permanently blocking pain signals by freezing the cell. Both treatment modalities are effective in managing chronic pain and inhibiting the transmission of pain signals.

Our study is the first article in the literature that we could find that discusses the effectiveness of minimally invasive techniques in this refractory pain. This study aimed to compare the short- and long-term effects of RFT and CA on pain management and functional recovery to determine which method is more appropriate for which patient groups. Post-treatment pain levels (visual analog scale [VAS]), functional limitations (FD), activity limitations (FI), and ankle function (AOFAS) were analyzed, and the results were compared. This study is expected to provide clinicians and researchers with information on the efficacy of these treatment methods.

## 2. Methods

This retrospective study was approved by the Ethics Committee of Adana City Training and Research Hospital with the decision of the ethics committee approval date: 11.09.2024, approval no: 158. It aimed to compare the effects of RFT and CA on pain management and functional recovery, as well as evaluate the short- and long-term effects of the treatments.

A retrospective analysis of patient records was conducted to evaluate procedures applied to individuals experiencing pain lasting at least 6 months and unresponsive to conservative treatment approaches. A total of 97 patient files were accessed. However, due to a lack of data and follow-up, only 71 patient files were accessed. The remaining patients with complete follow-up and data were divided into two groups based on the procedures performed. However, the number of patients with complete data follow-up is not equal.

This study included 71 patients underwent RFT or CA for chronic pain treatment. There were 42 patients in the RFT group and 29 patients in the CA group.

### 2.1. Inclusion Criteria

Patients who underwent RFT or CA for chronic pain treatment.

Patients evaluated for the VAS, FD, FI, and AOFAS scores in the pre- and postoperative periods.

Patients with a follow-up period of at least 6 months after treatment.

### 2.2. Exclusion Criteria

Patients who have previously received the same treatments.

Patients with cerebral palsy (CP), polyneuropathies/peripheral nerve damage, muscle atrophies and motor neuron diseases, sacroiliitis/ankylosing spondylitis, and reactive arthritis/psoriatic arthritis.

Patients who were not followed up for 6 months after treatment.

Patients with infection at the treatment site.

Patients with hemostasis disorders.

The main evaluation criteria of the study focused on pain management and functional improvement. The efficacy of both treatment modalities was evaluated using the following parameters:

VAS: It is used to measure the patients' pain level and is graded between 0 and 10.

FD (functional disability score): It is used to measure the limitations experienced by patients in their daily activities.

FI (activity limitation score): It is used to evaluate the patient's ability to perform physical activities.

The American Orthopedic Foot and Ankle Society (AOFAS) score: It is used to evaluate foot and ankle functions.

The patients' pain VAS, FD, FI, and AOFAS values were measured in the preoperative preparation room before the procedure.

RFT and CA are commonly used for pain treatment [5]. Heel spurs are treated using not only conventional treatments (e.g., nonsteroidal anti-inflammatory drugs, shoe insoles (shoe pads), corticosteroid injection into the lesion, orthoses, exercise, and night splints) but also innovative treatments (e.g., radiotherapy, extracorporeal shock waves, and percutaneous and/or intralesional RF application), which have been preferred in recent years. Calcaneal spur excision is frequently accepted as the last option, particularly in persistent and recurrent cases [4–7].

### 2.3. Intralesional Thermal Radiofrequency Therapy (RFT)

RFT has been used to reduce the pain level of patients and accelerate functional recovery [8, 9]. Sır and Eksert concluded that applying RFT to the lesion itself is sufficient in patients with calcaneal spurs [10]. Furthermore, RFT can be used to neutralize the transmission of pain signals by heating the nerve tissue. In the present study, intralesional RFT was used owing to its easy and shorter application technique.

### 2.4. Intralesional CA

In situ destruction of the lesion in painful tissues via freezing is well known in many branches of surgery. Ultrastructural studies have demonstrated that CA induces the formation of ice crystals within the cells and that the resulting cell damage is osmotic and secondary in terms of the chronological development of tissue necrosis [1]. Intralesional CA was used in the CA group.

Furthermore, CA freezes the nerve tissue, thereby inhibiting the transmission of pain signals. During the CA treatment, cryogenic techniques ensure that the pain signals are permanently blocked [11, 12].

CA is aimed at providing long-term pain management and functional improvement. This technique, which has no systemic side effects, causes minimal tissue damage [13].

### 2.5. Application Technique

Patients were taken to the pain management room, which was equipped with an anesthesia machine, monitor, fluoroscopy, and RF devices. They were placed in the prone position on the surgical table, and pulse oximetry, electrocardiogram, and noninvasive blood pressure were monitored according to the recommendations of the American Society of Anesthesiology. An intravenous cannula was placed, and 1 mg of dormicum was administered for sedation.

**Figure 1:**
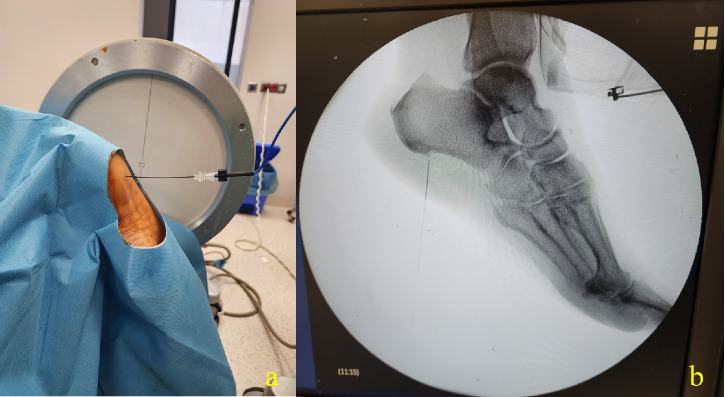
Application photo of radiofrequency procedure. (a) Placement of the RF cannula in the patient lying in prone position, (b) lateral fluoroscopy image of the RF cannula.

**Figure 2:**
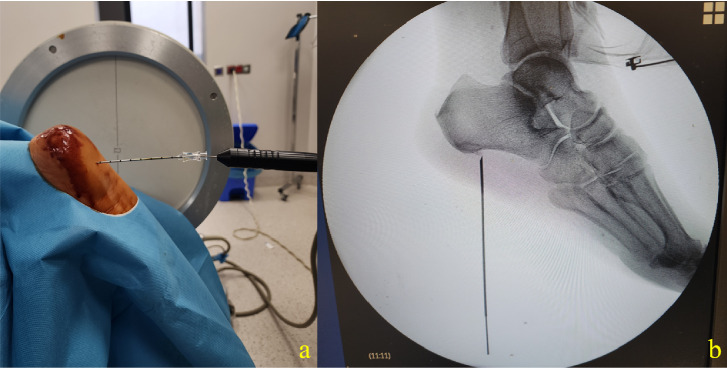
Application photo of cryoablation procedure. (a) Placement of the CA cannula in the patient lying in prone position, (b) lateral fluoroscopy image of the CA cannula.

The procedure site was then disinfected with a povidone–iodine-based solution and draped. Fluoroscopy was used to identify the calcaneal suture, providing a lateral view. The skin was infiltrated with 2 mL of 2% lidocaine at the insertion point, and the needle required for insertion was inserted under fluoroscopy guidance. Needle positions were then considered successful if sensory stimulation was positive with concordant pain at 50 Hz and < 0.5 mV and negative for any motor stimulation up to 2 Hz and 2 mV. In the RF group, thermocoagulation was applied at 80°C for 60 s (Figure 1). Patients in the KA group were frozen at −70°C for 60 s using a cryogenic probe after localization was confirmed (Figure 2). No complications occurred during the treatments, and the patients were discharged 1 h after the procedure.

Data were collected through pre- and postoperative clinical evaluations and follow-up examinations. The pre- and postoperative pain levels, functional limitations, as well as foot and ankle function were recorded at 15 days, 1 month, 3 months, and 6 months. Furthermore, the patients were regularly followed up throughout the treatment process.

The collected data were statistically analyzed to compare the results of the two treatment groups. The effects of RFT and CA on the VAS, FD, FI, and AOFAS scores were evaluated using an independent two-group *t*-test. The SPSS software (Version 25.0) was used for statistical analyses, and *p* < 0.05 was considered to indicate statistical significance.

## 3. Results

The effects of intralesional RFT and CA on pain management, functional limitation, and ankle function were evaluated pre- and postoperatively. The findings were detailed to understand the effects of both treatment modalities at different time periods.

**Figure 3:**
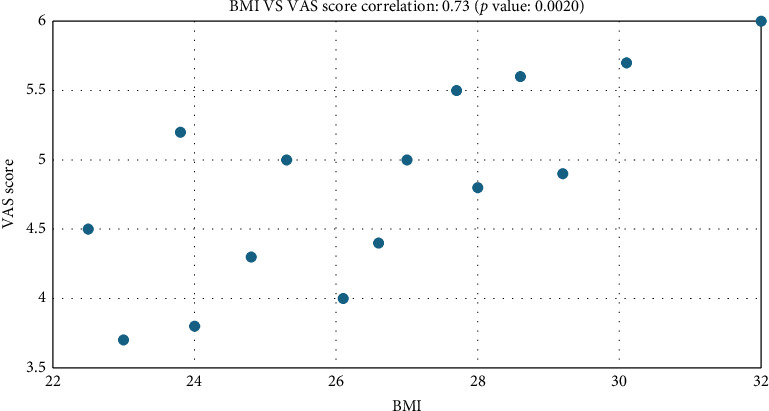
BMI and VAS (pain score) correlation.

The age of the study participants ranged from 28 to 52 years, and their BMI values were between 22.3 and 31.1 (Figure 3). The gender distribution was balanced in the RFT and CA groups (Table 1).

Furthermore, no significant differences were observed in the demographic characteristics between the groups. This suggests that the treatment results were not affected by the demographic characteristics of the patients and that the treatment modalities were comparable.

The VAS score was used to measure the pain reduction after treatment. The VAS and AOFAS scores were examined as pain and functional scores before and 1 and 6 months after the procedure, respectively. The VAS has a numerical rating of 0–10 [14].

**Figure 4:**
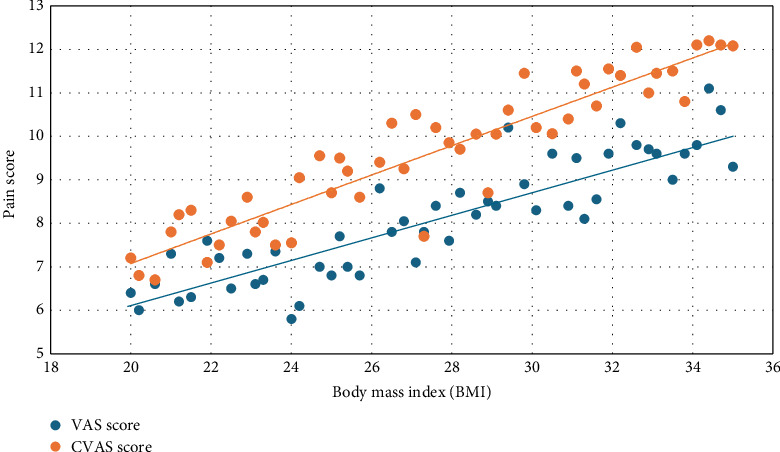
Correlation between BMI and pain scores (VAS/CVAS). This figure demonstrates the positive correlation between BMI and pain scores (VAS and CVAS). Higher BMI values are associated with higher pain levels in both measurement types.

At 15 days, 1 month, 3 months, and 6 months, RFT was found to be more effective in managing pain than CA. Particularly at 6 months, the RFT group showed significantly lower VAS scores (*p*=0.00002). In the short term, RFT endowed the patients with faster pain relief, whereas CA induced slower pain control (Figure 4).

Foot Function Index (FFI): This index was developed to evaluate the impact of foot pathology on function in terms of pain, disability, and activity limitation. FFI is a 23-item index divided into three subscales. FD is a subgroup of FFI and consists of nine questions; it is evaluated over 90 points in total [15].

**Figure 5:**
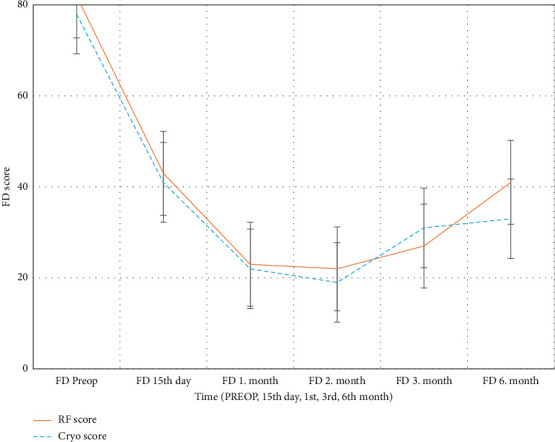
FD scores over time (RF vs. Cryo). Line graphs with error bars (mean ± SD) illustrating the temporal change in functional disability (FD) scores in radiofrequency thermocoagulation (RFT) and cryoablation (Cryo) groups. The RF group demonstrated consistently greater improvement in FD compared to Cryo.

The FD score was used to evaluate the functional limitations experienced by the patients in their daily activities. At 15 days, 1 month, 3 months, and 6 months, the RFT group had lower FD scores than the CA group (15 days, *p*=0.0045; 6 months, *p*=0.0011). Furthermore, RFT reduced the functional limitations more rapidly and effectively than CA and enabled the patients to return to their daily activities sooner after treatment. Compared with the RT group, the CA group exhibited a slower decrease in functional limitations and higher FD scores until the sixth month (Figure 5).

The FI score was used to evaluate the limitations of patients in performing daily activities. It is a subgroup of the FFI and contains five questions and a total of 50 points [16, 17].

**Figure 6:**
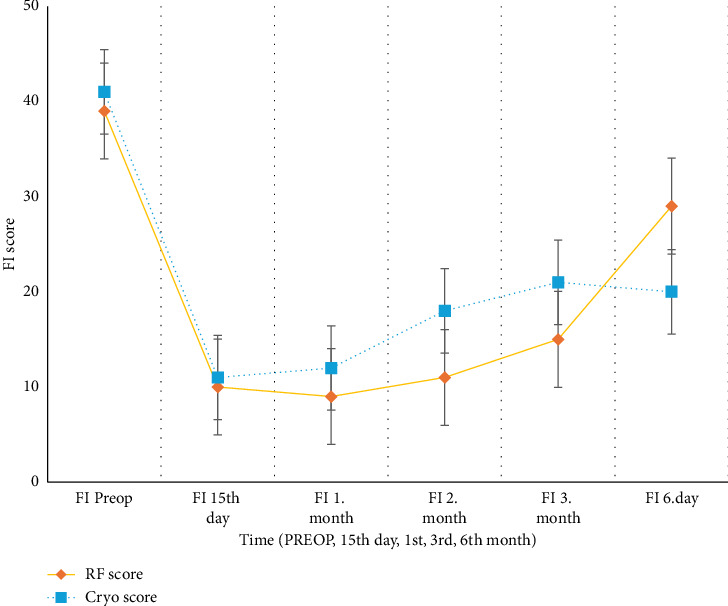
FI scores over time (RF vs. Cryo). Line graphs with error bars (mean ± SD) depicting the changes in functional index (FI) scores across follow-up periods (preoperative, 15th day, first month, third month, and sixth months). RF treatment resulted in superior functional recovery compared to Cryo.

The RFT group showed faster improvement in FI scores at 15 days, 1 month, 3 months, and 6 months (15 days, *p*=0.0171; 6 months, *p*=0.0171). Although the FI scores in the KA group improved over time, they remained higher than those in the RFT group. This indicated a slower recovery in the activity limitations after CA. At 6 months, the patients in the RFT group were able to continue their daily activities with fewer limitations (Figure 6).

The AOFAS score is one of the most important parameters for assessing foot and ankle function. The AOFAS score is graded on a scale of 0–100 points; values below 70 are considered to indicate poor results; values between 70 and 79, average results; values between 80 and 89, good results; and values between 90 and 100, excellent results [18].

At 15 days, 1 month, 3 months, and 6 months, the CA group had higher AOFAS scores. This indicates that CA is more effective than RFT in terms of functional improvement in the long term.

**Figure 7:**
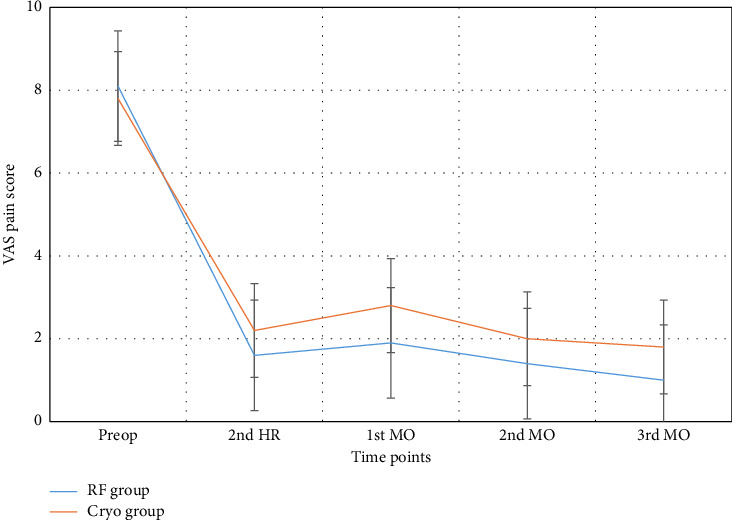
Temporal change in VAS scores following RF and cryoablation. VAS scores decreased significantly over time, especially in the RF group. This figure illustrates the superior long-term pain control of RF.

Particularly at 6 months, the CA group had significantly higher AOFAS scores than the RFT group (*p*=0.0001). This indicates that CA is more capable of improving ankle function in the long term (Table 2, Figure 7).

**Figure 8:**
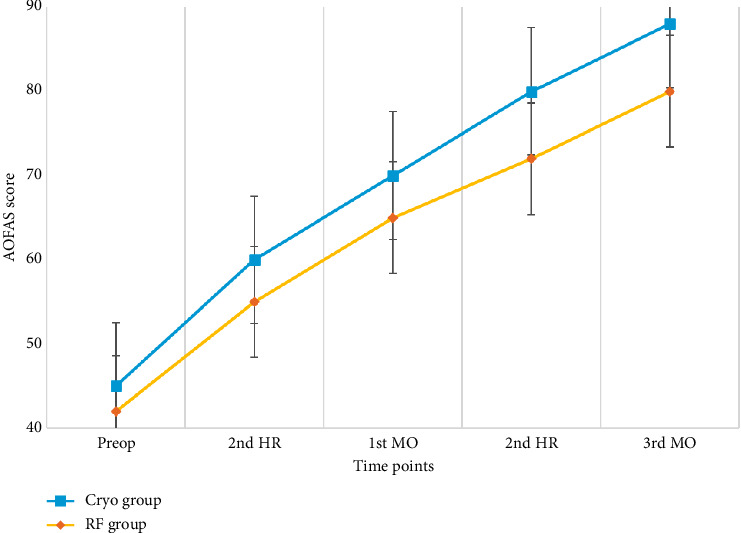
AOFAS scores over time (RF vs. Cryo). This figure compares functional recovery over time using AOFAS scores. The RF group showed higher functional improvements at all time points.

**Figure 9:**
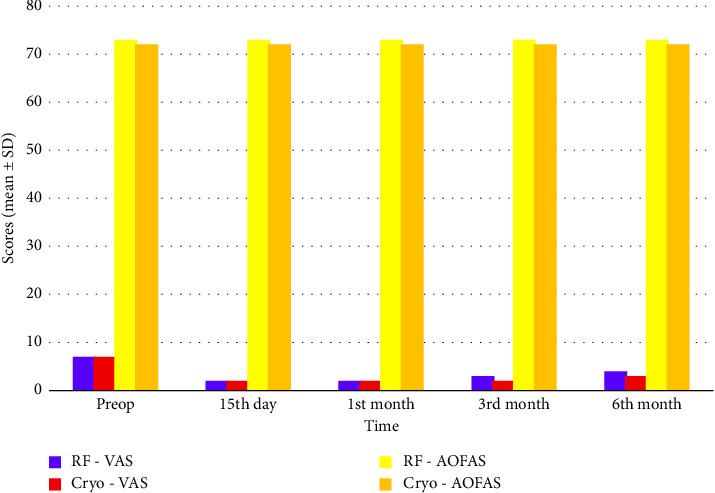
VAS and AOFAS scores over time (RF vs. Cryo). This bar chart compares the final outcome scores at the third month postprocedure. RF outperforms Cryo in all metrics with lower pain and better functional outcomes.

The findings of this study indicated that RFT was more effective than CA in managing pain in both the short and long terms. As regards functional improvement (AOFAS score), CA yielded superior results in the long term. At 6 months, the CA group achieved better foot and ankle function. However, in terms of FD, RFT enabled patients to return to their daily activities sooner and with fewer limitations, and the CA group had longer recovery process (Table 3, Figures 8 and 9).

## 4. Discussion

This study thoroughly examined the short- and long-term effects of intralesional RFT and CA concerning pain management, functional limitations, and functional recovery. Intralesional RFT has shown superiority over intralesional CA regarding short-term pain treatment. The VAS scores swiftly diminished in the RFT group during the initial 15 days post-treatment and remained considerably reduced for up to 6 months. The results suggest that each therapy modality possesses distinct benefits; however, the selection of treatment should be tailored to the patient's recovery objectives. The results are thoroughly examined below.

This study included 71 patients aged 28–52 years (BMI, 22.3–31.1). Of them, 64.8% were women and 35.2% were men. These data support the relevant articles in the literature. Basdelioglu, in an article examining CS risk factors in 420 patients, found findings supporting our results. He found that CS is more common in women than in men and that the risk of developing CS increases with increasing BMI, particularly in patients with a BMI > 30, with the highest risk [19]. In general, a positive correlation was observed between the BMI and VAS scores. Particularly, as the BMI value increased, the pain scores and functional limitations also increased (Figure 1). However, the correlation coefficient was moderate, and this relationship was not always evident (*r* = 0.35).

In the literature, there are no studies comparing intralesional thermal RFT and CA for the treatment of calcaneal spurs. In their study, Tas and Kaya performed ESWL and RFA on 236 CS patients and, as a result of the comparison, found RFA to be more effective in improving function scores than the ESWL group. They concluded that RFT was more effective, especially in patients with limited activity. These findings support our results in patients who underwent RFA [20]. In their study, Yapici et al. applied steroid injections, ESWL, and RFA to 229 patients with plantar fasciitis but found no significant superiority among the treatments. They reported that all three treatment methods provided significant pain reduction and that RFA could be recommended for the patient groups we applied in our study, especially for patients resistant to other treatments [21]. In another study, Kurtoglu et al. examined the effectiveness of RFA and found significant changes in pain and function in 261 plantar fasciitis patients, and presented it as an alternative to surgery [22]. Erdoğan et al. compared platelet-rich plasma and RFA. This treatment, applied to 96 patients, showed similar reductions in pain scores in both groups. While not included in the PRP group in our study, the significant reduction in the RFA group supports our results [23]. Pulsed radiofrequency (PRF), a RF procedure, can be used in the treatment of calcaneal spurs. This method, which provides a temperature increase of up to 42 degrees Celsius and helps modulate pain pathways without causing tissue damage, has been used in the literature for the treatment of calcaneal spurs. Eke et al., in a retrospective study of 460 patients, observed an increase in AOFAS scores and a decrease in pain scores with PRF, similar to the results in our study [24]. However, these improvements cannot be compared due to the different procedures and treatment alternatives employed. As supported by these studies, RFA is an effective and minimally invasive treatment option that does not cause serious complications.

CA is a widely used neurolysis technique in the treatment of chronic pain. Literature reveals that it is widely used safely for chronic pain syndromes such as lumbar facet joint pain, SI joint pain, post-thoracotomy syndrome, temporomandibular joint pain, chronic knee pain, phantom limb pain, neuropathic pain, and abdominal pain. It can be applied to the appropriate area after a response is elicited from the source of the pain via sensory stimulation or local anesthesia [5]. Goyal et al. examined 425 patients in their review and reported that CA provided a significant reduction in pain scores for up to 6 months, but they could not reach sufficient findings in publications with longer follow-up [25]. Naskar et al. emphasized in their published review that CA is safer than many neurolysis methods and is effective in chronic pain conditions [26]. However, Biel and colleagues, when listing the contraindications for CA, specifically recommend against its use in patients with cryoglobulinemia, cold urticaria syndrome, and Raynaud's syndrome, unlike RFT. They also noted that hypopigmentation, depigmentation, and alopecia may occur, particularly in the superficial nerves of the application area. They also noted that numbness may occur for up to 30 s after application. In our study, none of our patients experienced these symptoms, and no permanent side effects were observed [27]. In their study, Truong et al. examined CA, RFT, and placebo in 120 facetogenic chronic low back pain patients as a single-blinded, randomized controlled trial, but they could not find any superiority over placebo; however, in our study, both methods showed a significant decrease in both pain palliation and other scores [28].

Overall, the RFT treatment was more effective in terms of pain management and short-term improvement. It provided the patients with faster pain relief and enabled them to return to their daily activities sooner. However, the CA treatment was found to be superior to the RFT treatment, particularly in terms of long-term functional improvement and recovery of ankle function.

### 4.1. Limitations

This study has several limitations that should be acknowledged. First, the relatively small sample size (*n* = 71) may limit the generalizability of the findings and reduce the statistical power to detect subtle effects between treatment groups. Second, the retrospective design inherently introduces risks of selection bias, incomplete data capture, and a lack of standardized follow-up intervals. Third, the absence of a control or placebo group precludes definitive causal interpretations and limits the ability to differentiate treatment effects from natural disease progression or placebo responses. Lastly, individual variability in pain perception, functional adaptation, and response to intervention may have influenced the outcome measures, particularly in subjective scales such as VAS and FD. Future prospective, randomized controlled studies with larger cohorts are necessary to validate and expand upon these findings.

## 5. Conclusion

This study aimed to compare the short- and long-term effects of RFT and CA on chronic pain management and functional recovery. The findings of the study indicated that both treatment modalities have different advantages and may be more appropriate for different patient groups under certain conditions.

RFT rapidly reduced pain in the short term and enabled patients to return to their daily activities sooner. Furthermore, RFT more rapidly improved the patients' VAS (pain score) and FD (functional limitation score) than CA, and this difference persisted until 6 months. These findings suggest that RFT is a suitable option for patients seeking rapid pain management and functional improvement in the short term.

Meanwhile, CA was found to be superior to RFT in terms of functional improvement in the long term. According to the AOFAS scores, patients receiving CA treatment showed higher functional results up to the sixth month. CA may provide a more lasting effect in the long term and may be effective in preventing pain recurrence.

These findings suggest that the treatment choice should be based on the needs of the patient. RFT may be preferable for patients seeking rapid pain relief and short-term functional improvement, whereas CA may be suitable for patients seeking long-term functional improvement and permanent pain control.

In conclusion, intralesional RFT and CA are effective treatment options for calcaneal spur cases in terms of pain management and ankle function recovery. Therefore, it is important to provide patients with personalized treatment according to their needs. This study provides important information on the potential benefits of intralesional RFT and CA in clinical practice and compares them with other treatment options.

## Figures and Tables

**Table 1:** Demographic and clinical characteristics of the study population.

Parameter	Value
Variables (*n*)	71
Age (years), mean ± SD	40 ± 12
Gender, *n* (%)	
Female	46 (64.8%)
Male	25 (35.2%)
BMI (kg/m^2^), mean ± SD	26.7 ± 4.4
Type of procedure, *n* (%)	
RFT	42 (59.2%)
CA	29 (40.8%)
Comorbidities, *n* (%)	Plantar fasciitis (19), diabetes mellitus (18), hypertension (17), none (17)
Prior treatments, *n* (%)	NSAIDs (15), orthotic insoles (14), corticosteroid injection (14), night splints (13), none (15)

**Table 2:** Comparison of AOFAS scores between RFT and CA groups at different time points.

Time point	AOFAS score (mean ± SD)-RFT	AOFAS score (mean ± SD)-CA	*p* value
Pre-op	40.0 ± 3.2	42.0 ± 3.5	0.114
15 days	60.3 ± 4.1	61.1 ± 4.4	0.328
1 month	75.6 ± 3.8	73.4 ± 4.0	0.092
3 months	85.5 ± 2.6	86.7 ± 2.9	0.244
6 months	90.2 ± 2.1	91.0 ± 2.3	0.189

**Table 3:** Change over time for RFT and CA.

Time	VAS (RFT)	VAS (CA)	FI (RFT)	FI (CA)	AOFAS (RFT)	AOFAS (CA)	FD (RFT)	FD (CA)
Pre-op	7.9 ± 0.6	8.1 ± 0.5	50.0 ± 3.1	52.0 ± 2.9	40.0 ± 5.2	42.0 ± 4.7	95.0 ± 4.1	96.0 ± 4.3
15 days	2.1 ± 0.4	2.4 ± 0.5	20.3 ± 2.8	22.1 ± 2.4	60.3 ± 4.1	61.1 ± 3.6	50.3 ± 3.2	52.1 ± 3.4
1 month	1.9 ± 0.3	2.0 ± 0.4	15.6 ± 2.1	16.2 ± 2.0	75.6 ± 3.9	76.2 ± 3.7	40.6 ± 2.9	42.0 ± 3.1
3 months	1.5 ± 0.2	1.7 ± 0.3	10.5 ± 1.9	11.7 ± 1.5	85.5 ± 2.8	86.7 ± 3.0	30.5 ± 2.4	31.7 ± 2.6
6 months	1.2 ± 0.1	1.3 ± 0.2	5.2 ± 1.1	6.0 ± 1.2	90.2 ± 2.5	91.0 ± 2.3	20.2 ± 2.1	21.0 ± 2.0

## Data Availability

The datasets used and/or analyzed during the current study are available from the corresponding author upon reasonable request.

## Nomenclature

AOFAS: American orthopedic foot and ankle society
BMI: Body mass index
CA: Cryoablation
FFI: Foot function index
VAS: Visual analog scale
